# Adjuvant therapy for renal cell carcinoma, finally a new standard?

**DOI:** 10.3389/fonc.2022.926661

**Published:** 2022-09-29

**Authors:** Alex Renner, Carlos Rojas, Annerleim Walton-Diaz, Mauricio Burotto

**Affiliations:** ^1^ Medical Oncology Department, Bradford Hill Clinical Research Center, Santiago, Chile; ^2^ Department of Urology, Chilean National Cancer Institute, Urofusion Spa, and Department of Urology, Universidad de Chile, Santiago, Chile

**Keywords:** immunotherapy, renal cell carcinoma, adjuvant, pembrolizumab, tyrosine kinase inhibitions (TKIs) therapy

## Abstract

Localized renal cell carcinoma (RCC) has the potential to be cured with surgery alone; however, some patients have a high risk of relapse and may benefit from additional treatment. Several efforts have been made to identify effective strategies, with mostly negative results. However, recent results with immune checkpoint inhibitors may change the current standard, and several ongoing trials are exploring new alternatives. In this perspective, we aim to provide an overview of previous adjuvant therapy efforts, current data supporting the use of checkpoint blockade, and a future outlook for adjuvant therapy in renal cell carcinoma.

## Introduction

Approximately 431,288 new cases of renal cancer were diagnosed in 2020 worldwide (1). Patients with localized and locoregional diseases are potentially curable with surgery, and their 5-year overall survival rate is 93% and 71%, respectively (2). After surgery, observation has been the standard, as most trials of adjuvant therapy have failed to show a clinically meaningful benefit while generating significant toxicity. New strategies are certainly needed, and adjuvant immune checkpoint inhibitors are the most promising alternatives, having shown efficacy first in metastatic renal cell carcinoma (RCC) and now also positive results for the use of pembrolizumab after surgery in selected patients. These data may finally lead to a change in the long-held approach to locoregional RCC.

## Selecting patients for adjuvant treatment

Certainly one of the most critical issues for adjuvant treatment is patient selection, identifying which patients with localized disease are at increased risk of relapse and may benefit from treatment in addition to surgery. Both in the clinical setting and in clinical trials, the most utilized prognostic factor has been the tumor–node–metastasis (TNM) staging system. Several nomograms such as the Kattan, Leibovich, UCLA Integrated Scoring System (UISS), and SSIGN risk score have been developed, and interesting advances have been made with genetic recurrence scores. In spite of this, our capability to identify patients at high risk is still limited, and most trials we will review in this article have used solely the TNM score to define their high-risk populations.

## Adjuvant therapy with IFNα and IL-2

Around the year 2000, there were several efforts to identify potential improvements in outcomes for RCC patients with adjuvant therapy. Clark et al. (8) assessed high-dose bolus interleukin-2 (IL-2) given postoperatively in patients with high-risk renal cell carcinoma defined as completely resected locally advanced (T3b–4 or N1–3) or metastatic (M1) disease. Sixty-nine patients were enrolled, but the study was closed early as it proved futile, showing similar disease-free survival (DFS) for the IL-2 and placebo groups (p = 0.73). Another study, by the Eastern Cooperative Oncology Group/Intergroup Trial (9), compared observation to adjuvant interferon alfa (IFNα) in 283 patients after complete resection of locally extensive renal cell carcinoma defined as stage T3–4a and/or N, M0. Results showed a median overall survival (OS) of 7.4 years in the observation arm and 5.1 years in the treatment arm (p = 0.09). Later on, the combination of IFN and low-dose IL-2 was studied in the POLAR-01 study (10). It included 310 patients with completely resected pT2–3b pN0–3 M0 tumors. Results showed similar relapse-free survival (RFS) and OS, with an estimated hazard ratio (HR) of 0.84 [95% CI, 0.54–1.31; p = 0.44] and 1.07 (95% CI, 0.64–1.79; p = 0.79), respectively.

## Adjuvant therapy with VEGFR tyrosine kinase inhibitors

As the understanding of the pathogenesis of RCC advanced, it allowed for the development of antiangiogenic drugs, targeting the VEGF family of receptors (VEGFRs), such as sorafenib, sunitinib, pazopanib, and axitinib. They rapidly became the standard of care for metastatic RCC, after showing clinically meaningful improvement in outcomes for these patients. Exploring antiangiogenic drugs in the adjuvant setting was the next logical step, and five large randomized controlled trials (RCTs) were carried out to test their efficacy.

The ASSURE (11) study included 1,943 patients diagnosed with RCC who had completely resected tumor stage (T) of T1b or greater non-metastatic disease, including both clear cell and non-clear cell histologies. They were randomized in a 1:1:1 fashion to receive sunitinib, sorafenib, or placebo for 54 weeks. The primary endpoint was DFS as assessed by the investigator. After 1,323 patients had enrolled, a high discontinuation rate was observed (44% of patients on sunitinib and 45% of patients on sorafenib), and the protocol was amended to a reduced starting dose for both drugs. Results showed no significant difference in DFS, with a median DFS of 5.8 years for sunitinib (HR 1.02, 97.5% CI 0.85–1.23, p = 0.8038), 6.1 years for sorafenib (HR 0.97, 97.5% CI 0.80–1.17, p = 0.7184), and 6.6 years for placebo. Overall survival did not differ between groups; 5-year OS was 77.9% for sunitinib, 80.5% for sorafenib, and 80.3% for placebo.

Another study that evaluated the efficacy of adjuvant sunitinib was the S-TRAC trial (12). It included 615 patients diagnosed with non-metastatic locoregional RCC defined as T3 or T4 and N0 or Nx, or any T stage with local nodal involvement, and clear cell histology. They were randomized 1:1 to receive sunitinib or placebo for 1 year. The primary endpoint was DFS assessed by central review. Attrition was high in the sunitinib arm, only 56% of patients completed the full 1-year treatment and adverse events (AEs) were the main reason for discontinuation. The median DFS was 6.8 years in the sunitinib group and 5.6 years in the placebo group (HR 0.76, 95% CI 0.59–0.98, p = 0.03). Based on these results, the US Food and Drug Administration expanded sunitinib’s indication to include the treatment of patients at high risk for recurrence after nephrectomy. An OS analysis was later reported, where there was no statistically significant difference (ref S-TRAC OS), the median OS was not reached in either arm, and the HR for sunitinib versus placebo was 0.92 (95% CI 0.66–1.28; p = 0.6).

After the S-TRAC results were published, Haas et al. looked at the subgroup of patients within the ASSURE trial who had high-risk RCC features (13), namely, pT3, pT4, or node-positive. Out of the initial 1,943 patients in the ASSURE trial, 1,069 met those criteria. They did not find a significant difference for either DFS or OS. Five-year DFS rates were 47.7%, 49.9%, and 50.0% for sunitinib, sorafenib, and placebo, respectively, while 5-year OS was 75.2%, 80.2%, and 76.5% for the same groups.

PROTECT (14) is a study that compared adjuvant pazopanib versus placebo, in patients with resected RCC pT2 (high grade) or ≥pT3, including N1. A total of 1,538 patients were randomized, and the treatment duration was 1 year. The study was later amended to reduce the initial daily dose of 800mg to 600 mg. The primary endpoint was changed to DFS only in patients who received the 600-mg dose. Results showed no difference between the arms (HR 0.86, 95% CI 0.70–1.06 p = 0.165). Later, an overall survival analysis was published (15), and it showed no significant difference in OS between the pazopanib and placebo arms (HR 1.0, 95% CI 0.80–1.26, p > 0.9).

The efficacy of axitinib has also been evaluated in the adjuvant setting; the ATLAS trial (16) randomized 724 patients with pT2 and/or N+ resected RCC to receive axitinib or placebo, for a minimum of 1 year and up to 3 years. The trial was stopped due to futility, as the pre-planned interim analysis found no significant difference in DFS (HR 0.870, 95% CI 0.660–1.147, p = 0.3211). In the highest-risk subpopulation, defined as pT3 with Fuhrman grade (FG) 3 or pT4 and/or N+, they report a 36% reduction in DFS as determined by the investigator (HR 0.641, 95% CI 0.468–0.879, p = 0.0051) but not as determined by the independent review committee (IRC), which was 27% (HR 0.735, CI 0.525–1.028, p = 0.0704).

Another large trial which evaluated adjuvant tyrosine kinase inhibitor (TKI) treatment is the SORCE study (17), a three-arm design comparing 3 years of placebo, 1 year of sorafenib followed by 2 years of placebo, or 3 years of sorafenib. Patients included in the study had completely resected, clear cell, or non-clear cell RCC with a Leibovich recurrence score of 3 or more; the Leibovich score incorporates not only TNM stage but also nuclear grade and the presence of tumor necrosis. Due to the high discontinuation rate, the sorafenib dose had to be reduced from an initial 400 mg twice per day to 400 mg once daily. Also, the primary research question was changed in light of the primary results from the ASSURE and S-TRAC trials. The results for the SORCE study showed no difference in DFS between patients assigned to 3 years of sorafenib and those assigned to placebo (HR 1.01, 95% CI 0.82 to 1.23, p = 0.946).

It is significant to note that all TKI adjuvant trials suffered from a high discontinuation rate: 28% with sunitinib in S-TRAC, 36% with pazopanib in PROTECT, 44% with sunitinib and 45% with sorafenib in ASSURE, and 23% with axitinib (ATLAS) and over 50% with sorafenib in SORCE even considering the mentioned dose reduction.

## Immune checkpoint inhibitors

Drugs that block the activity of PD-1, PD-L1, and CTLA-4 immune checkpoint proteins have proven their efficacy in the metastatic setting on a wide array of tumor types. That includes first-line RCC, with pembrolizumab in combination with axitinib, pembrolizumab plus lenvatinib, avelumab in combination with axitinib, and nivolumab combined with ipilimumab, all currently approved by the Food and Drug Administration (FDA). It is also relevant that they have shown efficacy in the adjuvant setting for some tumors, with both pembrolizumab and nivolumab being approved for locally advanced melanoma following complete resection, nivolumab currently approved for the adjuvant treatment of patients with urothelial carcinoma who are at high risk of recurrence after radical resection, and atezolizumab for PD-L1 positive stage II to IIIA resected non-small cell lung cancer. Considering all this, there is a strong rationale to support studies looking into potentially improved outcomes for RCC patients treated with adjuvant immune checkpoint inhibition.

The Keynote-564 study (3) is a phase 3, double-blind, randomized clinical trial that recruited 994 patients diagnosed with RCC who were at high risk for recurrence after nephrectomy and compared adjuvant therapy with pembrolizumab 200 mg intravenously every 3 weeks or placebo for up to 1 year until disease recurrence or unacceptable toxicity. The definition for high risk of recurrence was tumor stage 2 with nuclear grade 4 or sarcomatoid differentiation, pT3 or higher, regional lymph-node metastasis, or stage M1 with no evidence of disease (NED) after resection. The primary endpoint was DFS according to the investigator; OS was a secondary endpoint. Baseline characteristics were similar between arms, the median age was 60.0 years for both, and 5.8% of patients in each arm had M1 NED, while PD-L1 combined positive score was ≥1 for 73.6% of patients on pembrolizumab and 76.9% of patients with placebo. The discontinuation rate was 20.7% for pembrolizumab and 2.0% for placebo, and grade 3–5 adverse events were identified in 18.9% and 1.2% of patients, respectively. The median duration of treatment was 11.1 months for both arms. Results showed that the risk of recurrence or death was 32% lower in the pembrolizumab arm than in the placebo arm (HR 0.68, 95% CI 0.53 to 0.87, p = 0.002), and this was consistent across subgroups. The percentage of patients who remained alive and recurrence-free at 24 months was 77.3% (95% CI, 72.8 to 81.1) with pembrolizumab and 68.1% (95% CI, 63.5 to 72.2) with placebo. These results led to the FDA approval of adjuvant pembrolizumab on 17 November 2021, certainly a landmark moment in the history of RCC. An update of this trial has been presented at ASCO GU (18) with a 30-month follow-up, showing that the risk of recurrence or death is 37% lower in the pembrolizumab arm than in the placebo arm (HR 0.63, 95% CI 0.50–0.80, nominal p < 0.0001).

Smaller studies have also been reported for neoadjuvant immune checkpoint inhibitors (ICIs). Gorin et al. (19) reported that three doses of neoadjuvant nivolumab were safe and tolerable in patients with non-metastatic high-risk RCC, and while all 17 patients had stable disease by RECIST, one patient (6.7%) demonstrated features of an immune-related pathologic response. The phase II Neoavax study (20) tested neoadjuvant avelumab plus axitinib prior to nephrectomy in 40 patients with cT1b–4cN0–1M0, grades 3–4, non-metastatic RCC; 12 patients (30%) had a partial response, median primary tumor downsizing was 20%, and importantly, no patient had a primary tumor progression during neoadjuvant therapy.

## Ongoing trials

There are several other checkpoint inhibitors currently undergoing evaluation in the adjuvant RCC setting. The IMmotion010 trial (18) will compare atezolizumab for 1 year of treatment to placebo for patients who have undergone nephrectomy and are at a high risk of recurrence (pT2 grade 4, pT3a grade 3–4, pT3b/c any grade, T4 any grade, or TxN+ any grade); M1 patients are also eligible if they have had complete resection of limited metachronous/synchronous metastasis with no evidence of disease. The primary endpoint is DFS.

Checkmate 914 (19) is evaluating a combined immunotherapy approach where both nivolumab and ipilimumab administered for 1 year will be compared to placebo in part A of the study, and then in part B, the same combination will be compared to a single drug nivolumab for 1 year and observation. Eligible patients are pT2a G3/G4, pT2b or higher any G, any T N+, and M1 with NED. The primary endpoint is DFS as assessed by BICR.

The RAMPART study (20) is a multi-arm multi-stage trial that is planning to enroll 1,750 patients and will start with three arms: observation, durvalumab for 1 year, or durvalumab for 1 year plus two cycles of tremelimumab, which is a monoclonal antibody against CTLA-4. Eligibility criteria are based on the Leibovich risk score and will include patients with intermediate risk (scores 3 to 5) and high risk (scores 6 to 11). The primary endpoints are DFS and OS.

PROSPER RCC (4) is a study sponsored by the National Cancer Institute (NCI). To enter this study, patients must have a renal mass consistent with clinical stage T2Nx, N+ any T, or M1 planned to be definitively treated such that the patient will be considered M1 NED. On one arm, patients will undergo nephrectomy, and on the other arm, patients receive one dose of nivolumab and then undergo nephrectomy 7–28 days later, followed by adjuvant nivolumab every 4 weeks for up to 9 cycles. The primary outcome is event-free survival (EFS).

A summary of the mentioned trials is provided in **Table 1**.

**Table 1:** Neoadjuvant ICI trials.

Study(clinicaltrials.gov identifier)	TNM stage	Active treatment (duration)	Planned enrollment	Primary endpoint	Start date	Primary completion
Keynote-564 (NCT03142334) (3)	pT2 G4 pT3 or greater any grade N+ any T any grade M1 with NED	Pembrolizumab (1 year)	994	DFS by investigator	June 2017	December 2020
IMmotion010 (NCT03024996) (4)	pT2 G4 pT3a grade 3–4 pT3b or greater any grade N+ any T any grade M1 with NED	Atezolizumab (1 year)	778	DFS by investigator	January 2017	May 2022
Checkmate 914 (NCT03138512) (5)	pT2a G3–G4 pT2b or greater any grade N+ any T any grade M1 not eligible	Nivolumab plus ipilimumab (1 year)	1600	DFS by BICR	July 2017	July 2024
RAMPART (NCT03288532) (6)	Leibovich score 3 or greater is used as inclusion criterion instead of TNM	Durvalumab, durvalumab + tremelimumab (1 year)	1750	DFS and OS	July 2018	July 2024
PROSPER (NCT03055013) (7)	cT2 or greater, any N cN1 or greater, any T M1 with planned resection	Nivolumab (9 months), one dose given preoperatively	766	EFS	February 2017	March 2022

NED, no evidence of disease; DFS, disease-free survival; OS, overall survival; EFS, event-free survival.

## Discussion

Based on current evidence, we would argue that the use of adjuvant pembrolizumab for 1 year has become the standard for some patients with a high risk of recurrence after nephrectomy. As the potential benefit of this strategy will be proportional to the risk to our patients, patient selection is crucial. The inclusion criteria on the landmark Keynote-564 trial have been discussed, and it is quite an ample one, ranging from localized pT2 tumor patients to metastatic patients who were resected with no evidence of disease. We can already see on the subgroup analysis that while all patients derive benefit, this benefit is numerically much higher for M1 patients (HR 0.29, 95% CI 0.12–0.69) than for M0 patients (HR 0.74, 95% CI 0.57–0.96) and could be a major driver for the positive results for the entire study population. This underscores that patient selection will continue to play a key role in identifying who will benefit from adjuvant therapy; we believe that the evidence is very strong for using adjuvant therapy for M1 NED patients, while M0 patients should be carefully selected, and we would consider this strategy for more advanced cases such as T3N+ and T4 tumors. Unfortunately, no biomarkers or gene signatures have yet been validated to help us make that decision in the context of adjuvant immune checkpoint inhibition. For now, we must still rely on the TNM staging and good clinical judgment.

Although most adjuvant trials have used DFS as their primary endpoint, we must also carefully consider overall survival data once they become available, especially since first-line therapy for RCC has advanced so much in recent years. Patients already exposed to an ICI agent in the adjuvant setting will have no clearly defined standard of treatment in the first-line setting. We hope that patients randomized to the placebo arm on these adjuvant ICI trials receive the appropriate standard of care therapy including an anti-PD1/PD-L1 agent in case of a systemic recurrence, in order to be able to identify the real OS benefit of moving current first-line agents to the adjuvant setting.

Finally, when selecting patients for adjuvant treatment, we must also consider two additional factors: toxicity and access. Although the safety profile of pembrolizumab is generally considered to be quite acceptable, we must note that 18.9% of patients suffered from grade 3–5 adverse events in the Keynote-564 trial, and some of them such as hypothyroidism and adrenal insufficiency are not reversible and will require lifetime treatment. Regarding access, due to its high cost, lack of overall survival data, and lack of a “value in cancer” evaluation, in many countries, these drugs are not funded by the health system and must be paid out of pocket, which generates a huge barrier for access to therapy.

With several ongoing trials in this space, we must expect current standards to be continually challenged, not only from other immune checkpoint inhibitors but also from other strategies including combination therapies and preoperative/postoperative dosing.

## Data availability statement

The original contributions presented in the study are included in the article/supplementary material. Further inquiries can be directed to the corresponding author.

## Author contributions

AR: Main manuscript writing. MB: Title and main topics to be covered, revision and approval. CR: Discussion. AW-D: Review from the urologic point of view, revision. All authors contributed to the article and approved the submitted version.

## Conflict of interest

The authors declare that the research was conducted in the absence of any commercial or financial relationships that could be construed as a potential conflict of interest.

## Publisher’s note

All claims expressed in this article are solely those of the authors and do not necessarily represent those of their affiliated organizations, or those of the publisher, the editors and the reviewers. Any product that may be evaluated in this article, or claim that may be made by its manufacturer, is not guaranteed or endorsed by the publisher.
